# Community codes of conduct as proactive intervention for toxicity in games

**DOI:** 10.3389/fpubh.2026.1842010

**Published:** 2026-07-14

**Authors:** Rachel Kowert

**Affiliations:** Department of Psychiatry, University of Cambridge, Cambridge, United Kingdom

**Keywords:** code of conduct, community guidelines, digital community, moderation, trust and safety, video games

## Abstract

So called “toxic behaviors” (e.g., hate speech, harassment, doxxing) are pervasive in online gaming communities, with research consistently documenting significant negative consequences for player wellbeing, community health, and industry revenue. While gaming studios and platforms have developed a range of reactive and proactive moderation strategies to address these harms, most existing approaches remain reactive, focusing on punishment after harm has already occurred. Community codes of conduct represent a foundational yet underutilized proactive intervention that has received comparatively little systematic attention. This literature review synthesizes existing research to address two core questions: (1) What is known about the design, implementation, and accessibility of community codes of conduct in gaming environments? and (2) How do codes of conduct influence player behavior? Drawing on a final corpus of 84 articles identified through systematic search of academic databases and supplemented by transparency reports from major gaming studios, the review maps the current landscape of both reactive and proactive moderation approaches before examining the specific role of community governance documents. Findings reveal significant gaps between current industry practice and evidence-based best practice. More than half of popular multiplayer games either lack accessible codes of conduct or bury them within legal documentation, limiting meaningful player engagement. Where codes do reach players, however, the evidence is promising: well-designed codes are associated with measurable reductions in toxic behavior, increased community engagement, and stronger community norms. Design features that enhance effectiveness include prominent placement and readability, values-centered and game-specific content, community-centered participatory development, transparent enforcement mechanisms, and integration of peer and community social influence. Despite these positive indicators, the evidence base remains limited, with most studies examining discrete platforms and short-term outcomes. This review concludes with evidence-based recommendations for industry practice, synthesized into the EFFECT Framework — a practical tool distilling six core evidence-based design principles for developing community codes of conduct that actively shape community culture. Together, these contributions call for greater research investment in how codes of conduct can be optimized as living community tools (rather than static legal documents) to meaningfully reduce toxicity and build resilient gaming communities.

## Introduction

Video games are the dominant form of entertainment in modern society, surpassing the movie and music industries combined in value (1). In June of 2025, the Entertainment Software Association reported that 205.1 million Americans regularly play video games in their daily lives (2), producing $59.3 billion in game and hardware sales in 2024 alone. This growth is not only representative of the commercial success of the industry but also signals a fundamental shift in online social interaction. For billions of people worldwide, online games have become a primary venue for connecting with others (3, 4). As social platforms (134), gaming communities offer playful spaces where people can maintain relationships with close friends and distant family members alike as well as forge new friendships with players from across the globe. Research shows that online gaming communities, and social gaming experiences generally, have a net positive effect on players social and psychological well-being (5–7), with 71% of players across the globe agreeing that video games are a “great way to meet new friends and make relationships” (8).

However, this transformation of gaming into burgeoning social platforms has introduced significant challenges around player behavior. Disruptive behavior (often referred to as “toxicity”) has become a persistent issue, with many players experiencing frequent instances of sexism, racism, and harassment during gameplay (9). While there is disagreement on the exact definition of what constitutes toxicity (10, 11), toxicity in online games generally encompasses behaviors that intentionally disrupt gameplay and negatively affect other players’ experiences (12). This can include trolling (13), griefing (14), and cheating (15), as well as more severe actions like hate-based harassment (16), doxxing, and stalking (11).

Nearly all players have witnessed some form of toxicity in their gaming communities (17–20, 135). In 2022, the Anti-Defamation League reported that four in five adults (86%) report experiencing harassment in online multiplayer games, representing over 67 million adult game players (ADL, 2022). Looking at the provenance of toxicity in game players across the United States and United Kingdom, researchers found 82% of players report being a victim of some form of toxicity, 88% reported witnessing it, with most of these experiences happening within the gaming spaces themselves (as opposed to game-adjacent our other out-of game social spaces; (18)). Recent research looking into *League of Legends* found that as many as 24% of players engage in some form of toxic behavior, with 70% of players’ matches being disrupted by some form of toxicity (21). While this is one specific example within a specific ecosystem, it illustrates the robust impact of toxicity’s prevalence within the in-game environment.

It is important to note that what is considered disruptive behavior, or “toxic,” can vary across games, regions, and even individuals within the same game and region, as different players and play groups have their own norms and tolerance for what could be considered toxic acts (22, 136). This is because community norms and social influence play a decisive role in shaping the prevalence and nature of toxicity in any given community as users to tend to conform to the norms of their community, adjusting their toxicity to match the toxicity levels of the communities in which they participate (23–25, 129).

Regardless of these variations in community norms and individual tolerance levels, research consistently demonstrates that exposure to toxic behavior produces significant negative outcomes for those who experience it, from short-term distress to long term post-traumatic stress disorder symptomology (9). Toxic gaming communities have also been found to lead players to disengage from the community itself, limiting their communication, hide identities or abandon games entirely (26–29). Persistent exposure to toxic behavior has been found to be associated with lower self-esteem, increased social isolation, and depressive symptoms (30–32).

Toxic gaming environments are also a financial cost to the industry, with most players saying they avoid playing certain games if it has a reputation for a toxicity environment (7 out of 10), temporarily or permanently leaving a game or platform because they were subjected to hate and harassment (6 out of 10), and choosing not to spend money in a game or platform because of how other players treated them in the community (6 out of 10; (128)). Research from the University of California Irvine found that players report they spend up to 54% less money in games with toxic communities (Steinkeuler, 2023), representing substantial revenue losses for an industry built on player engagement.

High-levels of toxicity also presents pervasive cultural challenges through the normalization of harmful behavior in gaming environments, creating a self-perpetuating cycle where victims often become perpetrators (16, 22, 34, 35). This can drive away players who would otherwise participate, further reinforcing the toxic culture and compounding financial loss.

In response to these challenges, the gaming industry has developed a range of interventions for combating toxicity, which can be categorized across five different categories: empowering (tools so they players can combat toxicity themselves), supporting (providing support to the player exposed to toxicity), priming (creating awareness of what is allowed or acceptable), sanctioning (punishment for breaking the rules), and detecting (automated detection of toxicity; (36)). These different methods for addressing toxicity can be further categorized in relation to when the intervention system is applied, either *before* (proactive) or *after* (reactive) harm has happened (36, 37, 125, 137). Reactive interventions are the most common, take place in response (i.e., in *reaction to*) to toxic behavior and are often intended to punish poor behavior, discourage repeated offenses, and, in some cases, to remove highly disruptive players from the space altogether (36). Conversely, proactive interventions are intended to prevent or rectify issues before the harmful interaction ever occurs. The most successful strategies are those that integrate both of these approaches into the ecosystem (37). These two approaches are discussed in more detail below.

### Reactive approaches to moderation

The most common reactive strategies to address toxicity in games include bottom-up player reporting systems (report, mute, block) and top-down content moderation from the gaming studio or platform.

Bottom-up strategies are those that are initiated by the players themselves. Nearly every console and gaming platform have the option for players to report disruptive players, which allows players to flag disruptive individuals or content for moderator review (38). This is a very common tool available across nearly every console and gaming platform, although it remains unclear how often people are using these tools and/or their effectiveness for combatting disruptive behavior in games.

While most players report that they do use reporting tools (85%; (17)), there is a general consensus that reporting tools are ineffective (at least partially due to their lack of transparency and consistency in enforcement; (17, 39, 40)), and are the least used resources used by bystanders and victims when faced with toxic interactions (40). Furthermore, even though reporting gives players more involvement with the moderation process by allowing them to police their own interactions, it also puts the onus of moderating on the player themselves. Reporting systems are important for short-term reductions in offensive behavior; however, reporting in and of itself is not effective and changing the behavior over time, the culture of the space, or bringing support to the victim of the behavior (33).

Muting and blocking tools also allow players to protect themselves from harassment by restricting communication with problematic individuals (15). Muting prevents direct communication with the problematic player in-game, while blocking additionally excludes them from matchmaking. These tools are typically integrated within broader reporting tool suites, giving players immediate self-protection options after harassment occurs. However, unlike reporting systems that can lead to broader community protection through moderation actions, muting and blocking only protect the individual user who activates them and do not prevent the problematic player from harming others in the gaming ecosystem (131).

These bottom-up tools are important, as they give individual players immediate control over their own exposure to toxicity. However, they are not effective at addressing the broader community impacts of disruptive players or toxic social environments. In response to these limitations, gaming studios and platforms have implemented top-down reactive approaches to remove problematic content and players from the broader ecosystem (i.e., “content moderation”). Generally speaking, these systems involve collaboration between detection systems that automatically flag disruptive content, identify the users causing description, and/or alert human moderators who review cases requiring more nuanced judgment (38, 41). Social media platforms have long been the primary innovators for this kind of top-down content moderation, developing the tiered content moderation system that has inspired the approaches that have been adopted by the gaming industry for both text-based and voice activated moderation approaches.

This kind of content moderation is an enormous task, as platforms must monitor millions of posts a day from millions of users across different regions, each with their own set of government regulations and cultural understanding of what constitutes offensive content. This work also takes a significant human toll, as moderators face daily exposure to potentially horrific content, with reportedly little mental health support from their employers (42–44). While newer models of artificial intelligence are growing more sophisticated (e.g., (45)), these detection systems still struggle to account for context within each case (46, 47).

Third-party service providers and academic researchers are currently working to improvise these models that are already in use (138, 139), by developing more sophisticated approaches that can recognize conversational nuance through specialized word libraries tailored to specific games and regions (among other things) to better understand situational context (140, 141). Meanwhile, companies continue innovating with additional tools to reduce the burden on human content moderation, including shadow banning persistent perpetrators to reduce their impact (48) and implementing prompts (i.e., nudges) that encourage users to reconsider potentially harmful content before posting (49).

### Proactive approaches to moderation

While reactive content moderation remains the dominant industry approach, gaming studios and platforms also utilize more proactive strategies to address toxicity in games from the top-down (platform side) and bottom-up (player initiated).

From the top-down, platform side, companies have started to utilize AI and machine learning models for automated detection and moderation to moderate content before it goes live onto a platform or gaming community (138, 139, 141–144). Given the vast resources needed to moderate their communities on a global scale (50), many game companies utilize automated systems to prevent the use of banned words or usernames before they are shared across the wider network. However, there seems to be little or no standardization exists for this kind of preventative, word-list based moderation within the gaming industry. Different studios and platforms use different prohibited word lists, allocate varying levels of resources to maintaining these systems, and update their lists at different intervals. The Anti-Defamation League has published a series of reports outlining the limitations that result from this lack of standardization, documenting inconsistent effectiveness in preventing hateful or antisemitic usernames across different gaming platforms (51, 52).

Despite their limitations, AI and machine learning models are proving to be successful in their preventative approaches. *Activision* recently published a case study reporting the effectiveness of their voice moderation integration with *Modulate*, that has allowed them to action offending players above and beyond player reporting and reducing exposure to toxicity among the wider community by as much as 50% (53, 54).

Many gaming companies and platforms have also developed bottom-up, player led preventative interventions. For example, honor and endorsement systems that incentivize positive player interactions (55, 126) and peer influence interventions that leverage social norms to encourage positive behavior (56).

Honor systems are believed to incentivize positive player interactions by allowing players to reward their peers for notable teamwork and communication (55, 57, 125, 145) by allowing players to select other members from the session for praise after a match has completed. However, the effectiveness of this type of approach is unclear (57, 145). Players themselves have noted several logistical issues with the implementation of this kind of system, including that the inability to endorse one’s friends incentivizes playing with strangers, using reports as part of the scoring process turns reporting into a tool for harassment, and the added incentive causes some distrust towards the motivations of other players (57).

Despite these implementation challenges with formal honor systems, the underlying principle of leveraging peer relationships to encourage positive behavior has shown promise in other forms. For example, researchers are starting to test peer-based interventions and its role in norm setting as a preventative approach to combatting toxicity in gaming spaces (56, 58, 59). Preliminary findings indicate that interventions encouraging positive social norms from peers can be a viable tactic for preventing further disruptive behavior. For example, Zsila et al. (56) presented new players with testimonials from “expert” players with adaptive strategies for dealing with toxicity. This resulted in greater usage of adaptive strategies (e.g., muting, reporting, exiting the match), decreased usage of maladaptive strategies (e.g., retaliation), and decreased rumination. Recently the Institute for Strategic Dialogue (ISD) called out the potential of peer-intervention in this way, noting that “at the community level, gamer-led resilience and peer support models show considerable promise. Specifically, peer mentoring within guilds, esports teams, or fan communities can normalize inclusive behaviors [(58); p. 25].

### Effectiveness of current approaches to moderation

While there are various approaches to moderation currently being implemented across gaming studios and platforms, their effectiveness is unclear. This is, at least partially, because data relating to disruptive behavior and intervention remains either proprietary or measured through player reflections. The ways in which we can openly assess the effectiveness of moderation intervention in games in more detail below.

#### Industry self-assessment (transparency reports)

Transparency reports provide valuable insights into platforms’ moderation approaches and enforcement outcomes, focusing on downstream enforcement metrics (e.g., how many violations were detected, punished, or appealed). While it is important to know these metrics, it also creates a critical gap in that companies can demonstrate that they catch and punish toxic behavior, but cannot show whether their approaches have successfully discouraged these behaviors from happening initially (it also does not report unaddressed disruptions).

In 2022, the European Union passed the Digital Services Act (60), a landmark regulation that requires online platforms (including gaming companies) to produce more structured moderation processes with transparent enforcement practices and non-discriminatory appeal options (61). Due to this regulatory requirement, all collected reports follow similar formats: mission statements emphasizing moderation importance, lists of employed moderation interventions with links to codes of conduct, and statistics on players caught and punished for toxic behaviors. However, approaches to developing these transparency reports vary significantly across studio and platform. For example, Nintendo’s report (128) is a streamlined, two-page document, where they link their community guidelines and code of conduct, describe their moderation system (player reporting and a moderation team to review content), list their two enforcement measures (deletion of content or application ban), and provide statistics counting the number of reports and enforcement procedures implemented during the previous year.

In contrast, other companies have gone into far greater detail regarding innovations they have made to improve their moderation systems. Take-Two Interactive details each step and the reasoning behind each decision of its moderation and enforcement path (147). Electronic Arts points to its Positive Play Charter as a more accessible code of conduct, as well as its piloting of a voice chat moderation system (Electronic Arts, 2024). Niantic produces an individual report for each of its games, while the other companies combine their data into a single report (148). XBox touts its new enforcement strike system that better educates players about its enforcement procedures, as well as its expansive set of parental controls and its training and assistance program for supporting its human moderators (150). Epic Games includes a separate section for “Fortnite Islands,” its creative builder mode where players can design their own games (149), reflecting Epic’s status as a metaverse platform and the additional considerations it must take into account when moderating a broader spectrum of user-generated content (127, 133). Notably, none of the reports compare results with previous iterations to suggest year-over-year improvement, and only Ubisoft provided in-depth explanations of the metrics and logic being used for determining moderation accuracy (151).

Overall, the statistics each company provides are intended to portray evidence of successful moderation and transparency regarding the process. However, these metrics primarily measure enforcement activity rather than prevention effectiveness.

#### Player perspectives on intervention effectiveness

While transparency reports provide companies’ perspectives on their moderation effectiveness, understanding how players actually experience these systems reveals significant gaps between industry intentions and user reality. Player experiences reveal significant skepticism toward current industry moderation strategies, highlighting gaps between company intentions and user perceptions of effectiveness.

The effectiveness of any moderation intervention is fundamentally undermined by widespread player distrust toward gaming companies and their moderation systems, particularly reporting mechanisms (17, 38, 62–65). Players who have experienced moderation actions report confusion about enforcement processes and frustration with perceived inconsistency, stemming from a lack of transparency within moderation processes (64). While moderation at a global scale is an enormous task and social media companies have long faced similar criticism (66), it is vitally important for players to understand why they were punished in order for them to improve their behavior in the future (38). Many also question companies’ commitment to effective moderation, viewing enforcement as potentially conflicting with business interests since punished players are also paying customers (67).

This distrust is compounded by systemic implementation flaws that players readily identify. For example, *League of Legends* players admit to using reporting systems for poor play rather than actual violations, treating them as social tools rather than moderation mechanisms (38). They recognize that reporting works only retroactively and cannot prevent toxicity, requiring pairing with proactive tools like muting. Players also question companies’ moderation goals given the potential monetary implications of punishing paying customers - a concern supported by research suggesting toxicity positively correlates with purchasing behavior (67).

Players also respond differently to various moderation approaches, challenging one-size-fits-all strategies. Reid et al. (68) found that while players most readily use traditional tools like reporting and blocking, they also rate support-oriented interventions (positive messages, mood-boosting features) as effective (particularly among women). It may be that individual coping preferences significantly influence tool adoption (30) with many players employing a diverse set of interpersonal strategies and functional tools when experiencing toxicity (69).

Even when players appreciate innovative approaches, they identify implementation problems. Players responded positively to personalized matchmaking systems that filter out users with prior bans or problematic language patterns, but worried about isolating toxic players (potentially worsening their behavior) and creating longer queue times (70). Similarly, while testing auditory toxicity detection, players noted that victims defending themselves were incorrectly flagged, and automatic muting penalties disrupted legitimate strategic communication mid-match (71).

### The role of community codes of conduct

Community guidelines and codes of conduct are a less explored proactive approach to reducing toxicity in gaming spaces. These self-governance documents function as a hybrid intervention model as they are typically created by platforms using top-down processes but rely on both platform enforcement and peer-driven compliance (and enforcement, through player reporting) for their effectiveness.

The theoretical foundation for understanding why community codes of conduct are effective proactive strategies comes from decades of research on collective action and self-governance. Ostrom’s (1990) (152) and Ostrom’s (72) groundbreaking work on how communities successfully manage shared resources can give us some insight. In her work, she identified eight universal design principles for effective collective action: clearly defined group boundaries, rules tailored to local conditions, participatory rule-making, community-selected monitors, graduated sanctions, conflict resolution mechanisms, recognition of the rights to organize, and nested governance for larger systems (72, 152). Ostrom found that self-organized, community-driven governance consistently outperformed externally imposed rule systems when these core design principles were applied. That is, she found that parameters for how a community should behave are effective at driving behavior, particularly when they are self-organized and developed.

Gaming communities face similar collective action challenges to those Ostrom studied. Players share common gaming spaces and must coordinate behavior to maintain positive community experiences, while managing the persistent threat of disruptive individuals who can undermine the collective good. Just as fisheries or forest management required community members to develop and enforce sustainable practices, gaming communities need governance systems that can establish and maintain behavioral norms that serve the broader community interest.

Gaming communities have been informally making these rules across game, platform, and individual guild since the onset of online gaming. However, community codes of conduct also come more formally from gaming studios and platforms, providing formal guardrails that establish the expected social norms within any given community and create consistent reference points for acceptable (and unacceptable) behavior.

These guidelines help establish the *norms* in a community. Social norms, and in particular community-specific norms, are a powerful driver of collective behavior. For example, research into bystander intervention demonstrates that community attitudes can influence an individual’s moral behavior more strongly than their own internal moral compass (or moral cognitions; 153). Due to social expectations, individuals feel social pressure to behave and respond in alignment with the norms of their environment (73–75). This social pressure effect can be so strong that research has found a direct link between perceived community approval and bystander behavior in the face of online harm. That is, when bystanders perceive their friends as approving of cyberbullying, they report higher social pressure to join in the harmful behavior and consequently are more likely to participate themselves (75). Community codes of conduct are important and can help shape and formalize the expectations in any particular group.

Before moving forward, it is important to note that there can be some confusion around the distinction between “codes of conduct” and “community guidelines.” It could be argued that these documents serve different functions, with codes of conduct focusing on governance and behavioral restrictions (rules) while community guidelines provide more aspirational guidance for ideal behaviors (expectations and norms; (76)). However, it is also possible that codes of conduct and community guidelines are functionally equivalent documents that vary only in contextual framing (e.g., codes of conduct being more common in competitive gaming environments and community guidelines in cooperative ones).

Beyond terminological differences, there can also be a philosophical divide about what these documents should contain. Some believe that codes of conduct should take restrictive approaches that focus on prohibited behaviors and punitive consequences, whereas community guidelines take more aspirational approaches that emphasize positive community values and desired behaviors (77, 78). However, this either/or approach may be misguided as players recognize the difference between injunctive norms (what people think should be done) and descriptive norms (what people actually do), with both positive and negative behaviors existing on a spectrum of acceptability across different gaming communities (136). As such, the most effective governance documents would likely address both dimensions by establishing clear boundaries around prohibited actions while also promoting positive community standards.

Regardless of their specific label or philosophical approach, these documents serve as proactive forms of behavioral design intended to shape player behavior (24, 79), and while evidence suggests their effectiveness is conditional on design and implementation (80), the most effective versions likely incorporate both restrictive and aspirational elements. However, given both the lack of formal terminological distinction and the evidence supporting integrated approaches to code content, this review will use the phrase “*community codes of conduct*” as an umbrella term encompassing the front-facing governance documents used by companies and organizations to inform users of the types of behaviors expected or prohibited when using their product or service (81). These documents differ from other documentation like End User Licensing Agreements or Terms of Service as they are not legal agreements between copyright holders and users and are often written in a more easily accessible and concise way. Community codes of conduct have a long history across sectors, being originally applied in physical-world scenarios like corporate offices (132), open source projects (82), and scientific conferences (81). Today, they are utilized to set standards for virtual spaces like social media sites (83), livestreams (154), and online games (155).

Community codes of conduct could be considered the first line of preventative defense against toxicity, as players are typically presented with them before gaining access to the game and most gaming companies routinely require players to acknowledge these documents, creating apparent consent to behavioral standards (81). These codes can serve multiple functions: communicating expected behaviors to users, providing guidelines for moderation decision-making (particularly community moderation), and (often) specifying platform and community values.

Community codes of conduct can operate at the platform level or be customized for sub-communities of any size or type and generally consist of several different components: the behaviors that are acceptable for users to exhibit when in the space, warnings regarding actions and behaviors that users are forbidden to enact, and potential enforcement procedures if the code of conduct is ignored or broken. While these codes and/or guidelines are not necessarily intended to be “tools of discipline”, nor do they need to categorically list all possible scenarios, they are more generally intended to “*…give guidance and set boundaries for productive behavior, rather than exhaustively defining possible infractions and dictating specific punishments*” (84).

Today, critical gaps exist in our understanding of whether players actually engage with these materials and whether exposure translates to improved community behavior. Studies examining whether community codes of conduct actually improve behavior have produced mixed results across industries (79, 85, 86, 132, 156).

### Current project

Despite their foundational role in gaming platforms’ moderation ecosystems, fundamental questions remain about the nature and effectiveness of community codes of conduct in shaping player behavior. This literature review aims to clarify these gaps in our understanding by examining the existing research to address two key research questions:

**RQ1**: What do we know about community codes of conduct, their design, implementation, and accessibility?

**RQ2**: How do codes of conduct influence user behavior in gaming environments?

By synthesizing research on both the structural characteristics of effective codes and their measurable behavioral impacts, this review aims to provide evidence-based recommendations for developing governance documents that can meaningfully contribute to more gaming communities with lower rates of disruptive behavior (i.e., toxicity) and higher rates of resilience (i.e., effective self-governance to mitigate toxicity) in gaming spaces.

## Methods

The literature search for this literature review was conducted between September and October 2025. The list of search terms was developed prior to any data collection in order to control for bias. Searches were first conducted using the following academic databases: *Scopus*, the *ACM Digital Library*, and *IEEE Xplore*. Additional relevant citations were also taken from the references sections of articles found within these initial searches. Journalistic articles from mainstream news outlets or company press releases were included when identified through the same keyword searches and provided directly relevant content. The search terms used for literature regarding codes of conduct included (*games* OR *“online games”* OR *“video games”* OR *“multiplayer games”)* AND (“codes of conduct*”* OR *governance* OR *terms of service* OR *end user licensing agreement* OR *privacy policy)*. The terms used for moderation interventions included (*games* OR *“online games”* OR *“video games”* OR *“multiplayer games”)* AND (*intervention* OR *content moderation).* Inclusion criteria included studies discussing codes of conduct and their impacts on user behavior (particularly in regard to games), as well as proof-of-concept designs and evaluations of interventions designed to reduce toxicity in games and gaming spaces.

Given that codes of conduct are occasionally embedded within other forms of documentation (and that not all platforms maintain a dedicated code of conduct, with many instead relying on terms of service, end user licensing agreements, or privacy policies to serve an analogous governance function) literature regarding terms of service, privacy policies, and end user licensing agreements were also included in the search strategy. This decision was made to maximize coverage and ensure relevant work was not excluded on the bases of document nomenclature rather than function.

In addition, seven transparency reports from AAA gaming studios were added to the corpus. These companies are required by the European Union’s Digital Services Act (60) to produce reports on their moderation efforts. Such documentation was included in this review to provide insights into each company’s perspective on how effectively their interventions are able to combat toxicity on their platforms.

Once completed, the searches resulted in an initial corpus of 106 articles. To identify work of particular relevance, the abstracts of each article were examined and filtered out irrelevant work using the following set of exclusion criteria. Research examining player coping strategies for toxicity but not in direct relation to intervention tools were removed. Articles discussing terms of service, end user licence agreements, and privacy policies in relation to games but without discussion of player behaviors or conduct were also removed. Studies solely focused on the design of games intended to produce positive, collaborative play experiences were excluded. While pro-social game design is a promising and vital form of intervention (87), the literature on designing positive and collaborative games is distinct from community codes of conduct and therefore were excluded from this review.

After removing duplicates and works that did not meet the inclusion criteria, the final data corpus consists of 84 articles, with 10 regarding codes of conduct and 73 regarding interventions. Seven additional articles were included in this review that did not appear in the initial literature search (24, 76, 88, 89, 129, 136). Of these additions, Sherrick et al. (136) and Jiang et al. (90) were published after the initial literature search and the other four focused on codes of conduct in online forums rather than gaming specifically (24, 88, 89, 129). Tran (76) did not emerge in the literature review but does include information directly relevant to this review. As the content of these articles are directly relevant to the review’s scope they were added to the corpus. The full literature list can be found in Appendix A. All articles were read in full and synthesized for common themes, as detailed below.

## Results

The review of the literature revealed a complex landscape where community codes of conduct represent a foundational but underutilized intervention for addressing toxicity in gaming communities. The findings can be organized into three key areas that build upon each other:

- How do players currently engage with community codes of conduct?
- What design principles make these documents more effective?
- Do community codes of conduct influence player behavior in meaningful ways?

These three areas were identified inductively through thematic analysis of the reviewed literature, reflecting the recurring patterns and conceptual groupings that emerged across the body of work.

The following sections examine each of these areas in detail, highlighting current limitations and evidence-based opportunities for creating more effective community codes of conduct.

Taken together, the evidence demonstrates significant challenges in current implementation practices, particularly around player engagement and accessibility, while also identifying promising design approaches that could dramatically improve code effectiveness. Across all areas, the research reveals significant gaps between academic findings and industry practice, suggesting considerable untapped potential for improving community governance in gaming spaces by applying evidence-based approaches to the development and enforcement of community codes of conduct.

### Player engagement with community codes of conduct

It is difficult to directly assess the extent to which players engage with community codes of conduct. The most direct assessment in the research literature comes from the work of Böhme and Kopsell’s (91) who examined the patterns of more than 80,000 users and found that users spend approximately 8 s reading through end user license agreements (EULA) pop ups when presented upon login. While this study examined EULAs rather than codes of conduct specifically, it provides baseline data for understanding player interaction with legal and governance documents in gaming contexts. This 8-s reading time indicates that users are not likely to be meaningfully processing this kind of information but rather clicking through agreements without engagement.

Looking at games specifically, Grace et al. (155) examined the accessibility of conduct for 60 popular multiplayer games, in terms of document availability and findability. Their click test revealed that 13 of the 60 games (22%) completely lacked codes of conduct on company websites, while 19 others (32%) had codes buried within other governance documents like EULAs and Terms of Service. This means that more than half (54%) of the titles they examined lack accessible codes or place significant barriers for players to even have the opportunity to engage with these documents.

These findings are further supported by a large-scale automated analysis of codes of conduct across 9,586 multiplayer titles on Steam, which found that only 350 games (approximately 4%) had a retrievable, valid code of conduct, with availability concentrated among high-profile, well-resourced titles averaging 10 times more player reviews than the typical multiplayer game (90). This suggests that the accessibility barriers identified by Grace et al. (155) are not isolated to a small sample but reflect a systemic pattern across the broader gaming marketplace.

Beyond basic access, Grace et al. (155) identified patterns in how companies structure codes that may impact engagement levels. Through content analysis, they found that while companies typically include core elements (e.g., values, prohibited behaviors, reporting procedures) the variation in presentation and specificity suggests inconsistent approaches to engaging players. Some community codes of conduct provide specific examples from their games for increased clarity, while others rely on generic language that may not resonate with their specific player communities.

Player trust in codes of conduct can also serve as an important indicator of meaningful engagement. Several studies document widespread player distrust toward moderation processes due to lack of transparency (38, 63, 64, 92), which can include a lack of transparency within the community code of conduct. When players lose confidence in enforcement systems, their motivation to engage with the underlying codes can decrease and reduce participation in community governance processes. This distrust can stem from several sources: confusion regarding moderation decisions, frustration with perceived inconsistency across cases, and questions about companies’ moderation goals given potential monetary implications of punishing paying customers.

Engagement with the community codes of conduct can also be measured indirectly through behavioral indicators such as reporting rates, appeal submissions, and community discussion participation. That is, we can understand the extent to which players read, understand, and enact the rules and values presented in these codes through the ways in which they behave in the community. The research findings in this area are mixed. Some evidence suggests limited engagement or understanding as researchers have documented the misuse of reporting systems, with players flagging peers for poor play rather than actual code violations, indicating that engagement with codes may not be translating to understanding their intended purpose (38). For example, in interviews with *League of Legends* (Riot) players, Kou and Gui (38) found players openly admitting to using reporting systems as social tools rather than moderation mechanisms, recognizing that reporting only works retroactively and cannot prevent toxicity.

Other research suggests that community codes of conduct can be effective behavioral guides. Kowert et al. (93) found that community codes of conduct were better predictors of toxicity rates than top-down moderation efforts alone, even across different communities within the same gaming platform. This indicates that some player groups do engage with these documents and follow their guidance to self-regulate through peer enforcement. Additional research is needed to understand why player communities respond differently to community codes of conduct across gaming spaces and communities.

Taken together, the extent to which players engage with community codes of conduct remains unclear, largely due to inadequate measurement of their engagement patterns with these documents. Available research reveals significant variability in how players interact with governance information generally, from spending only 8 s reading end user licence agreements (EULAs) (91) to misusing reporting mechanisms for purposes other than their intended function (38). However, comprehensive data remains lacking on fundamental questions: how long do players typically spend reading codes when they access them? Do players understand the content they have reviewed? How does engagement vary across different player demographics? These gaps leave us with limited understanding of how effectively codes reach their intended audiences.

This measurement gap represents a significant limitation for both understanding and improving the effectiveness of community codes of conduct. Without clear baseline data on current engagement patterns and their variability, companies cannot systematically develop or test approaches for increasing meaningful player interaction with these governance documents. The available evidence suggests engagement ranges from minimal to potentially meaningful, but the lack of systematic measurement prevents us from understanding what factors drive these differences or how to optimize for better outcomes.

### Evidence-based design principles for community codes of conduct

Research into design principles for successful self-governance and collective action has a long history in the research literature. As aforementioned, Ostrom (1990) (152) and Ostrom (72) outlined eight universal design principles for successful collective action: clearly defined group boundaries, rules tailored to local conditions, participatory rule-making, community-selected monitors, graduated sanctions, conflict resolution mechanisms, recognition of the rights to organize, and nested governance for larger systems.

Contemporary research on gaming community governance seems to have independently arrived at similar conclusions. Researchers have identified six interconnected design elements for effective community codes of conduct that reflect Ostrom’s foundational principals: accessibility requirements, evidence-based content principles, community-centered development, community-based social influence, context-specific considerations, and transparency and trust-building. This convergence suggests universal patterns of how human cooperation and collective action can succeed across different contexts, whether managing common pool resources (like fisheries and forests as discussed in Ostrom’s work) or governing online gaming communities. Both research streams also show that self-organized, community-driven governance consistently outperforms externally imposed rule systems when these core design principles are applied. This research is discussed in more detail below.

#### Accessibility requirements

The accessibility of a community code of conduct is critical as research reveals significant barriers to code effectiveness stemming from poor accessibility. In this context, accessibility means the ability to easily and readily find the community code of conduct and understand its contents.

Accessibility is central to the guidelines for community codes of conduct outlined by Tran (76). In her model, she notes six universal guidelines for developing community codes of conduct: Clearly defined, publicized, stable, just, impartial, and timely. Two of these six relate to their accessibility: clearly defined (easy to understand) and publicized (easy to find and see).

Research has found that community rules that are more accessible and understandable are more likely to be adopted from new members (94). The focus on adoption from new members is important as newcomers who struggle to learn community norms are more likely to drop-out (this has been documented across different online spaces; (95)). This means community codes of conduct should use casual, game-specific language and place codes prominently on websites rather than burying them in legal frameworks.

Looking at games specifically, Grace et al. (155) found that most multiplayer games they assessed lacked accessible community codes of conduct, either because the codes were not easily found on company websites or because they were buried within dense legal documents like EULAs and Terms of Service. This creates a two-fold accessibility problem: players cannot easily locate the documents, and when they do find them, the highly technical legal terminology makes them difficult for average gamers to parse (157, 158). Additionally, codes embedded within legal frameworks may not even be legally enforceable.

Compounding this issue, codes of conduct are significantly harder to locate than other governance documents such as privacy policies, which typically appear in website headers or footers. Unlike privacy policies, codes of conduct lack standardized placement conventions, making them effectively invisible to the majority of players who do not actively seek them out (90).

The extremely low engagement rates documented earlier (91) suggest that traditional approaches requiring lengthy document review before gameplay are fundamentally flawed. Effective code design must therefore prioritize both physical access (i.e., findability) by ensuring prominent placement on websites rather than burial within legal frameworks and cognitive access (i.e., readability) by using casual, non-legal language with game-specific examples that players can relate to their own behavior.

#### Evidence-based content principles

Many organizations have published guidelines about what kinds of content of community codes of conduct should include (e.g., (96, 97)). The esports non-profit organization AnyKey (96) suggests that community codes of conduct should integrate core values, such as compassion (appreciate diversity), integrity (following the rules and taking responsibility), respect (respecting others), and courage (moderating your own and others behavior). They recommend that these values be translated into actionable frameworks through signable contracts distributed to all community members, with signing being mandatory for participation.

The Fair Play Alliance and Anti-Defamation League (97) make more specific recommendations like using terminology and actions native to the game, be brief and use direct examples of positive or negative behaviors to help players align with community expectations, explaining the consequences for not following the guidelines, and collaborating with communities when developing or updating codes.

Empirical evidence supports the idea that specific content approaches can increase community engagement. Fang et al. (24) found that effective community rules are those that articulate basic values, encourage moral behavior, and clarify restricted content or behavior. Notably, the researchers had originally hypothesized that greater content restrictions would lead to lower levels of user interactions; however, content restrictions showed no negative relationship with user interaction or posting metrics. As the researchers explained, “*Interestingly, rules within this category are often the most strict and restrictive, such as specifically disallowing certain times of content and media or enforcing verification of information sources. It is possible that these restrictions do indeed demotivate users from participating in the community, but also increase user interaction from preventing bad behavior between members*” (p. 16). The researchers suggest that content restrictions are likely interpreted as clarifying rather than constraining, helping to define community values and encourage participation by providing clear expectations. As previous research has shown, people can only contribute effectively to a community once they understand its needs, and clarifying what is allowed or disallowed increases the likelihood of meaningful member contributions (159). Importantly, having more rules was also not associated with decreased user participation, indicating that comprehensive guidelines do not create barriers when properly structured. This work is supported by other work in the space that found the most robust and broader rule sets were associated with communities with the least amount of disruption in their community (Smith et al., 2021; 70).

The most effective community codes of conduct are likely to be those that address both descriptive norms (what people actually do) and injunctive norms (what people think should be done; (76, 136) by actively promoting positive behaviors as expected community standards rather than just prohibiting negative ones. In this sense, community codes of conduct should function less as legal documents and more as community charters that articulate shared values while providing practical guidance for implementation. This requires moving beyond traditional “don’t do this” frameworks toward “here’s how we build the community we want together” approaches that actively shape both what people do and what they believe should be done in gaming spaces.

#### Community-centered development

Research has found that community-created rules based on existing norms (i.e., context-dependent) and values are the most effective for behavior regulation as well as adoption by new members (72, 76, 88, 98).

This effectiveness stems from collective action principles: when people organize to enforce their own rules, they are more intrinsically motivated to abide by them than externally imposed regulations (72, 99). This is, at least partially, because when communities create their own governance structures, they develop “psychological ownership” by exercising control over the rule-creation process, developing intimate knowledge of their communities’ context and needs, and investing personal energy and values to the outcomes (99). This ownership transforms compliance from an external obligation to an internal motivation, as community-created rules become part of a members’ “extended self” rather than imposed constraints. Psychological ownership over the development of one’s community code of conduct is likely to facilitate knowledge exchange within communities and adherence to the guidelines (99, 100).

Some gaming companies have begun experimenting with community participation in game design, including trust and safety efforts. Runescape has historically used polling and feedback systems to inform designers about potential improvements and community concerns (101), demonstrating how participatory design can shape gaming communities. In 2024, Roblox (102) established their Teen Safety Council initiative, where young users provide input on safety features and policies. Discord has also engaged community moderators and server administrators in developing moderation tools and guidelines (103).

This community-centered approach aligns with the aforementioned guidelines from the Fair Play Alliance and Anti-Defamation League (97), which emphasize collaboration with communities when developing or updating codes, recognizing that community buy-in significantly enhances adherence.

These approaches could be extended to developing and updating community codes of conduct. Current industry practice typically does not engage communities in this way as most community codes of conduct rely on top-down approaches that may not reflect community values or needs. This disconnect between generic corporate policies and specific community contexts undermines codes’ potential effectiveness in shaping behavior within particular gaming environments.

It is important to recognize that participatory design requires asking players to take active roles in moderating their communities. Successfully implementing this approach requires companies to develop specific guidance, infrastructure, and ongoing player support, which are resources that companies are not always willing or prepared to provide (155).

#### Community-based social influence

Beyond participatory design, research reveals the power of structured social influence in code effectiveness. Zsila et al. (56) found that presenting new players with testimonials from “expert” players resulted in greater usage of adaptive strategies for dealing with toxicity, decreased maladaptive responses, and reduced rumination. This effectiveness likely stems from the unique social bonds that players form through guilds, clans, regular play groups, and shared in-game experiences (104, 105). Friendship, social proximity, and the prosocial behaviors of one’s social network are among the strongest predictors of prosocial behavior (106–108), suggesting that incorporating voices of respected community members into codes can significantly enhance their impact by leveraging these existing social connections. These dynamics align with Cialdini’s (109) foundational principles of influence, particularly authority and social proof, which hold that people look to respected others and perceived consensus to guide their own behavior. Preliminary research supports this, as established, more-dominant users in the community (i.e., users with more authority) have been found to have a significant influence over the behaviors within their gaming spaces (37, 110, 111).

Rather than presenting rules as top-down mandates, effective codes can (and should) incorporate voices from respected community members (e.g., digital leadership) who serve as peer models for adaptive responses to community disruptions. When codes feature guidance from players that others know, consider in-group members, or feel connected to through shared community identity, the social bonds have the potential to activate prosocial motivations associated with friendship and group membership.

Current approaches primarily operate as static documents rather than dynamic community tools that leverage social proof and peer modeling to reinforce desired behaviors. Digital leaders like community managers and moderators can serve as critical norm-setters within their communities, using their positional authority to establish clear behavioral expectations and model appropriate intervention behaviors (42, 103, 111). By demonstrating how community standards should be upheld and that community members are expected to look out for one another, these leaders can cultivate environments where codes of conduct become living documents supported by peer enforcement networks.

#### Context-specific considerations

The context in which a community code of conduct is being developed needs to be considered. Effective code design must account for how different game features shape behavioral expectations. As discussed by Tran (76), they should be “just” or “reasonability fair for the space it is being applied in” (p. 74).

For example, research on gaming norms reveals that cooperative and competitive elements create distinct social dynamics (25, 40, 112, 136). For example, in multiplayer online battle-arena type games (MOBAs), communication itself can be perceived as hostile regardless of intent (160), suggesting that codes must account for genre-specific communication dynamics. Voice chat also presents particular challenges (130), as the presence of voice chat has been associated with a greater normalization of negative behaviors in gaming spaces than communities with only text-based communication (136). As such, a context-specific design consideration to integrate in communities with voice chat would be stronger, more specific guidelines and enforcement mechanisms for verbal interactions.

Research on Reddit’s ecosystem of community-created rules provides additional insight into context-specific design. Fiesler et al. (88) found that rule types vary significantly based on community subject matter rather than structural features like size or age. For example, art communities were most likely to have rules about formatting (85%) and copyright (56%), while politics communities had higher rates of rules about hate speech (67%) and trolling (56%). This suggests that effective community codes of conduct in games should adapt to game-specific risks and dynamics rather than applying universal templates.

These findings echo the points brought forward in Ostrom’s work which found that successful self-organized governance emerges when rules are “crafted to take local conditions into account” [(72), p. 150] and develop context-specific rules that address particular environmental and social challenges, rather than relying on externally imposed universal standards. For gaming communities, this would mean that community codes of conduct should be tailored to specific gaming spaces (e.g., mechanics, player demographics, and/or interaction patterns) rather than applying one-size-fits-all approaches.

Large-scale empirical analysis of codes of conduct across gaming platforms supports this need for context-specific design. Jiang et al. (90) found substantial variation in how specifically codes of conduct reflect individual games’ interaction and safety contexts, with community-focused genres such as MMORPGs producing significantly more context-specific governance language than genres featuring more transient player interactions. Notably, gameplay-mechanic-related violations showed the highest levels of specificity, while interpersonal harms (including harassment and hate and discrimination) showed the lowest, suggesting that the violations most likely to affect player wellbeing are also those most poorly addressed by current context-specific design practices.

The absence of context-specific design in gaming codes of conduct creates a fragmented landscape where communities fail to address their unique dynamics and challenges of their specific games and platforms.

#### Transparency and trust-building

As discussed earlier, player distrust in reporting systems and other trust and safety tools is often linked to transparency challenges around enforcement decisions and community standards (17, 38, 62–65). Ostrom’s research demonstrates that successful self-governance relies heavily on monitoring and conflict resolution mechanisms, transparent processes that build trust between community members and governance systems (72). As such, improving transparency around what is allowed and what is not could lead to an increase in player trust, engagement, and the effectiveness of community codes of conduct.

Transparent reporting pipelines in games have been shown to increase user trust in community governance systems (92). In relation to community codes of conduct, this could translate into the addition of explanations as to why a player has triggered an infraction, providing an opportunity to educate users about community norms and reduce future infractions from previous offenders (89). A key component of transparency is also the use of graduated sanctions that depend on the seriousness and context of the offense, ensuring that enforcement responses are proportional and predictable (72). In gaming contexts, this translates directly to the need for clear, transparent enforcement and decision-making processes with escalating consequences. Community members need to understand not just what the rules are, but how violations will be handled and how sanctions escalate based on severity and repeat offenses.

Some scholars have questioned whether current moderation protocols, as they are currently designed, are even sufficient for developing trust and transparency between game companies and their players (65, 113, 114, 161). Xiao et al. (65) point out that both victims and perpetrators of toxicity want greater transparency and control in the process – victims want avenues for repairing the harm they have experienced and perpetrators want clearer pathways to improve.

Sparrow et al. (114) frame a similar perspective around the top-down rulings of AI moderation tools, articulating that the “opaque nature of AI decision-making processes” often leaves “players feeling that the criteria used for decisions, which often had severe consequences such as bans or permanent account removals, were not clearly communicated” (pp. 344:21).

To be clear, meaningful transparency (not just disclosure) is what is needed to foster trust (115–119). Meaningful transparency is transparency that builds trust and enables accountability.

As discussed by Vogelgesang (120), transparency from leadership is characterized by “sharing relevant information, being open to giving and receiving feedback, being forthcoming regarding movies and the reasoning behind decisions, and displaying alignment between words and actions” (p. 43). This distinction of meaningful transparency is important because it is what builds trust and enables self-correction, while superficial transparency can actually increase distrust by appearing to hide important information behind technical disclosures. In gaming contexts, this means moving beyond transparency reports that simply list numbers of actions taken, toward explanations that help players understand the standards, predict enforcement, and feel confident that the system operates fairly and consistently.

Effective code design should therefore include clear explanation of consequences, detailed appeal processes, transparent decision-making procedures, and visible graduated sanction systems. However, many current industry practices fall short of meaningful transparency. Gaming companies’ transparency reports (e.g., Xbox, Microsoft, Riot, Roblox) often mirror problematic social media approaches (121), revealing varying enforcement approaches without sufficient detail for users to validate effectiveness claims or understand how sanctions are determined and applied.

### Impact and effectiveness of community codes of conduct

Current research on community codes of conduct effectiveness reveals promising but limited evidence for their impact on player behavior and community health. While studies demonstrate positive outcomes, significant gaps remain in our understanding of how and why codes work.

The most compelling evidence comes from studies measuring behavioral changes following code implementation. Kowert et al.’s (93) study of Minecraft servers found that server rules appear to matter more than moderation enforcement in shaping communication norms, demonstrating that well-designed codes proactively combat toxicity. Similarly, Feng et al. (24) found that proactive moderation approaches, including clear and effective codes of conduct, create healthier community environments by establishing norms that reduce unwanted online behavior. Codes of conduct can directly influence community members’ actions while serving as important reference points for reinforcing community values and guiding preemptive intervention from community moderators (162).

Contrary to concerns that rules might discourage participation, codes of conduct enhance player engagement. Fang et al. (24) examined different rule categories in online forums and found that both community values-centered rules (79% increase in user interaction) and content restriction rules (57% increase) positively correlated with engagement. Crucially, having more comprehensive rules was not associated with decreased participation, indicating that well-structured guidelines enhance rather than hinder community involvement.

Research also demonstrates that codes work best when integrated with broader community strategies rather than functioning as standalone documents. Honor systems that reward positive behavior become more effective when grounded in clear community values established through codes of conduct (55). However, implementation challenges exist: inability to endorse friends can discourage social play, and using reports in scoring systems can turn reporting into a harassment tool (57).

Despite these positive findings, the evidence base remains limited. Most studies examine specific platforms or game types, making generalizability unclear. Additionally, research focuses primarily on immediate behavioral outcomes rather than long-term community health or sustainability. More comprehensive studies are needed to understand which specific code design elements drive effectiveness and how different gaming contexts influence outcomes.

## Discussion

This literature review examined proactive and reactive moderation strategies for combating toxicity in gaming spaces, with particular focus on the development and efficacy of community codes of conduct. While few formal studies have been conducted to evaluate codes of conduct within this realm, the available data suggests that, in their current states, community codes of conduct have the potential to significantly influence player behavior positively. The research reveals promising indicators: codes that do engage players show measurable behavioral improvements, increase community engagement, and work effectively when integrated with broader proactive strategies.

Currently, codes of conduct represent a foundational but underutilized and understudied proactive intervention for addressing toxicity in games. The primary challenge lies not in their inherent effectiveness, but in current implementation approaches that limit their reach and impact. When accessible and well-designed codes do reach players, the evidence suggests they can establish positive community norms and reduce toxic behavior.

However, significant knowledge gaps remain. We have limited understanding of which specific design elements drive effectiveness, how different gaming contexts influence outcomes, or what implementation strategies maximize player engagement with governance documents. The research landscape reveals that we do not know what we do not know about optimizing codes of conduct for gaming environments.

This presents both a challenge and an opportunity. While current implementation practices may underutilize codes’ potential, the convergence of research findings with established principles of collective action suggests clear pathways for improvement through evidence-based design and community-centered development approaches.

### Moving forward: evidence-based recommendations for industry practice

The research reveals a clear opportunity: Community codes of conduct can effectively shape player behavior and reduce toxicity, but only when designed and implemented using evidence-based principles. Current industry practices significantly underutilize this potential, with most codes functioning as legal documents rather than community-building tools. There are several recommendations that can be drawn from the literature in how to develop more effective community codes of conduct in games. These are discussed in more detail below.

It is important to note that these recommendations are grounded in the synthesis of the literature as a whole rather than any single study. To help trace the research foundation for each recommendation, the thematic areas from which it draws are noted in parentheses.

#### Foundational requirements

*Prominent placement (Accessibility Requirements)*: Clear guidelines help users understand the community rules and boundaries, leading to more positive interactions and reduced conflicts, but only when users can locate and access them. If players cannot find community codes of conduct, they will not know what they are. This will inevitably lead to inconsistent enforcement and unclear community culture. The community codes of conduct should feature prominently in main community spaces and have multiple access points.

*Readability (Accessibility Requirements)*: Use casual, game-specific language and place codes prominently on websites rather than burying them in legal frameworks. While there are various guidelines, materials written at the seventh (122) or eight-grade reading level (123) are considered optimum for materials written for the public.

*Clarity (Accessibility Requirements; Evidence-Based Content Principles)*: Guidelines should clearly outline expected behaviors using active voice and concrete examples rather than abstract language or legal jargon. Clear, user-centric language that uses terms familiar to the audience can help facilitate comprehension and minimizes cognitive load. For example, abstract concepts like “maintain civil discourse” should be replaced with specific, actionable language like “avoid personal insults during gameplay.”

*Design for both norms and rules (Evidence-Based Content Principles; Context-Specific Considerations; Community-Centered Development)*: Address what players actually do (descriptive norms) and what they should do (injunctive norms) by promoting positive behaviors while establishing clear boundaries.

*Use comprehensive, values-centered content (Evidence-Based Content Principles; Community-Centered Development)*: Research shows that comprehensive and community values-centered rules increase engagement and do not create participation barriers when properly structured. Start with particularly the core, shared values and show how that translates into specific actions and boundaries.

*Enforcement transparency (Transparency and Trust Building; Context-Specific Considerations)*: Guidelines should clearly explain consequences and the process for addressing violations, including how reports are handled and what players can expect when rules are broken. This includes outlining escalation procedures (warnings, temporary restrictions, and permanent bans), timelines for moderation responses, and appeal processes. Players should understand not just what the rules are, but how they are enforced and what recourse they have if they disagree with moderation decisions. Without clear enforcement expectations, even well-written guidelines can feel arbitrary or unfair, undermining community confidence in the moderation system.

*Prioritize the most critical information (Accessibility Requirements, Evidence-Based Content Principles)*: Cognitive psychology research tells us that individuals can hold about seven (+/− 2) pieces of information in their working memory at any given time (124). Community codes of conduct should prioritize front loading the most important information and consider presenting in a tiered structure, making an easy to scan format that will allow users to understand the essential rules (3–5 items) at a glance but access more detailed information through expandable sections (with specific examples of each core rule) if desired.

#### Development process

*Community co-creation and ownership (Community-Centered Development; Community-Based Social Influence; Context-Specific Considerations)*: Involve the community in developing and refining guidelines to increase buy-in and cultural alignment. Engaging stakeholders (e.g., digital leadership, players) is important to ensure that guidelines are relevant, transparent and useful to those affected by them. This could include soliciting input during initial development, conducting regular community surveys about guideline effectiveness, creating feedback mechanisms for proposed changes, and establishing community advisory groups to help interpret guidelines in evolving gaming contexts. When players feel ownership over community standards rather than having them imposed from above, compliance and peer enforcement naturally increase while reducing the perception of arbitrary rule-making. Furthermore, community-created rules based on existing norms prove most effective for behavior regulation and member adoption.

*Leverage social capital (Community-Centered Development; Community-Based Social Influence; Context-Specific Considerations)*: Engage digital community leaders to evangelize and model community codes of conduct, transforming static rules into dynamic community tools that demonstrate how values translate into practice. This could involve identifying respected community members across different player groups (competitive players, casual gamers, newcomers, content creators, and influencers) to share positive examples through their content and interactions and/or provide feedback on how guidelines work in practice. This approach creates multiple pathways for guideline reinforcement beyond formal moderation.

*Design for gaming context (Evidence-Based Content Principles; Community-Centered Development; Context-Specific Considerations)*: Account for how different game features (cooperative vs. competitive, voice vs. text chat) shape behavioral expectations and adjust for this in your community codes of conduct. Competitive environments may require more explicit guidelines about acceptable trash talk versus harassment, while cooperative modes need emphasis on teamwork and constructive communication. Voice chat introduces real-time emotional expression that differs from text-based interaction, requiring guidelines that account for tone, interruption, and inclusive language practices. Different game genres also create distinct social dynamics. Guidelines should provide specific examples for each context: “In ranked matches, criticism should focus on gameplay decisions, not personal attacks”. This contextual approach can help players understand how core values apply across different gaming contexts.

#### Implementation strategy

*Integrate comprehensively (All six thematic areas)*: Building upon the foundational requirement of prominent placement (accessibility), community codes of conduct should be implemented holistically and comprehensively. This could include aligning these documents with pre-existing systems (honor systems, automated moderation, and peer influence mechanisms) while implementing multimodal delivery across various player touchpoints. Having a highly visible community code of conduct at multiple levels/areas of integration can enhance visibility and comprehension as it distributes the information across time and context.

*Some potential touchpoints*: onboarding flows, splash screens, contextual reminders when using specific game features (voice chat activation, competitive matchmaking), referencing appropriate guidelines when using nudging mechanics (“Remember: keep team communication constructive”), integration into reporting tools (“This behavior violates our respect guidelines”), and post-incident educational moments that connect violations back to community values rather than just listing penalties.

*Enable ongoing engagement (Accessibility Requirements, Community-Based Social Influence; Transparency and Trust Building)*: Develop systematic approaches for revisiting guidelines throughout the player lifecycle rather than one-time account creation review. Regular review and update of guidelines based on emerging issues and community growth is essential for maintaining effectiveness. This involves creating structured touchpoints that naturally integrate with player progression, such as seasonal reminders tied to new content releases, milestone check-ins when players gain certain privileges (moderator status, tournament participation), and refresher content during community events or policy updates. Ongoing engagement also includes providing opportunities for players to ask questions about guidelines in low-stakes environments, such as dedicated Q&A sessions, community discussions about guideline interpretation, and feedback systems that allow players to suggest clarifications or improvements. Rather than treating guidelines as static rules, this approach treats them as living documents that evolve with the community while ensuring all members stay informed of their current expectations and any changes.

*Measure and iterate (All six thematic areas)*: Develop systematic approaches to measuring both player engagement with codes and behavioral outcomes to ensure guidelines remain effective and relevant. Operationalize community engagement to organizational goals by tracking multiple indicators beyond simple activity measures (e.g., ecosystem health). This could include quantitative metrics such as guideline page views, time spent reading community codes of conduct, completion rates for onboarding materials, and behavioral indicators like report frequency, violation patterns, and appeal rates. Qualitative measurement should assess player comprehension through periodic surveys about guideline clarity, focus groups on community culture, and analysis of how players discuss rules in community spaces. Average session length and user-generated content rates provide insight into engagement depth, while tracking guideline exposure and positive community behaviors helps demonstrate effectiveness. Regular analysis should inform iterative improvements to both content and delivery methods, ensuring guidelines adapt to evolving community needs.

#### A framework for practice: the EFFECT framework

To distill and organize the core content and design principles identified across the findings within this paper and the recommendations above, the EFFECT Framework was developed as an evidence-based reference tool for practitioners. It synthesizes the design principles for what codes of conduct should contain and how they should be structured, providing accessible guidance for gaming studios, platform developers, and community managers seeking to move beyond compliance-oriented approaches toward governance documents that actively shape community culture (Table 1).

**Table 1:** The EFFECT framework.

Element	Principle
Easy to find and understand	Guidelines must be physically accessible (easy to find) and cognitively accessible (easy to understand), using everyday language rather than legal frameworks
Fact based content	Content should be grounded in evidence-based behavioral principles, addressing both descriptive norms (what people do) and injunctive norms (what people should do)
From the community	Participatory development creates psychological ownership, transforming compliance from external obligation to internal motivation
Everyone influences everyone	Leverage existing social bonds and peer modeling by incorporating voices of respected community members into governance documents
Context matters	Adapt guidelines to specific platform mechanics, game genres, and community dynamics rather than applying one-size-fits-all approaches
Trust through transparency	Clear, meaningful enforcement processes build community trust and enable accountability and self-correction

## Concluding thoughts

This literature review examined community codes of conduct as proactive interventions for addressing toxicity in gaming spaces, revealing both significant untapped potential and clear pathways for improvement. While these documents represent a foundational tool in gaming platforms’ moderation ecosystems, current implementation practices severely limit their effectiveness as behavior-shaping mechanisms.

The evidence demonstrates a fundamental disconnect between what research shows works and what the industry currently implements. Most concerning is the widespread player disengagement with governance documents. While it is unclear how many players meaningfully interact with this information, their capacity to do so and establish healthy (non-disruptive) community norms is severely constrained due to low accessibility and content clarity due to several systematic design flaws: codes buried in legal frameworks, written in inaccessible language, developed without community input, and treated as one-time compliance hurdles rather than living community documents.

However, the research also provides clear evidence for optimism. Studies demonstrate that community values-centered approaches can increase engagement while comprehensive, context-specific guidelines enhance (rather than hinder) community participation when properly structured. Discovering a clear convergence between design principles for developing effective community codes of conduct with established collective action principles reveals that there are universal patterns for effective community governance and supports the idea that the theoretical foundation for improvement is robust.

Perhaps the most significant finding is that the effectiveness of community codes of conduct are significantly impacted by community engagement, through community-centered design approaches and the social influence of digital community leadership. However, in order to understand the potential of community codes of conduct, we need to develop robust methodological approaches for measuring their effectiveness. Future research must develop frameworks that can assess the impact of community codes of conduct on long-term behavioral outcomes, including studies tracking behavior change and comparative studies across different code designs. Research could also explore how to optimize content and delivery of community codes of conduct as well as how codes can be adapted to different player types and gaming contexts while maintaining community coherence, potentially through modular structures or adaptive enforcement approaches. Additionally, future work should examine whether codes of conduct have differential effects across types of toxic behavior. The current literature treats toxicity as a broad construct, making it difficult to determine whether proactive governance documents are most effective at addressing norm-adjacent behaviors (i.e., ambiguous, context-dependent interactions that sit in the gray areas of community acceptability) versus more severe violations such as hate-based harassment or doxxing. Particularly interesting is whether codes of conduct occupy a unique intervention niche in these gray areas, where reactive enforcement mechanisms are least equipped to act. This remains an open and important empirical question.

As online gaming remains a primary social space for millions worldwide, the quality of these interactions shapes not only individual player experiences but broader social norms around digital citizenship. The business imperative is equally compelling: toxic gaming environments drive substantial revenue losses, while persistent toxicity creates self-perpetuating cycles that drive away potential players and further compound financial losses. The research reveals that effective toxicity prevention requires proactive community building rather than merely reactive punishment systems, an approach that serves both community health and business sustainability.

Today, critical gaps remain in measurement methodologies, highlighting the need for systematic approaches to evaluating code effectiveness and behavioral outcomes. The gaming industry must fundamentally shift the way they view these documents from codes of legal compliance towards understanding them as behavior-shaping tools that actively cultivate positive community cultures. However, the foundational work is complete: the gaming industry no longer needs to address these challenges through trial and error, as clear, actionable guidance now exists within the research literature.

## Data Availability

The original contributions presented in the study are included in the article/supplementary material, further inquiries can be directed to the corresponding author.
