# Supplementary material for: Community codes of conduct as proactive intervention for toxicity in games
Source: Front Public Health. 2026 Jul 14;14:1842010. doi: 10.3389/fpubh.2026.1842010 (PMC13407757; doi:10.3389/fpubh.2026.1842010)
Supplement: Supplementary file 1 [file Supplementary_file_1.docx]

Appendix A. Literature Corpus

Abramova, V., & Bernardino, J. (2013, October). Ethic in massively multiplayer online role-playing games. In 2013 1st International Conference of the Portuguese Society for Engineering Education (CISPEE) (pp. 1-9). IEEE.

Andrigueto, G. R., & Araujo, E. (2020, July). Fuzzy aggressive behavior assessment of toxic players in multiplayer online battle games. In 2020 IEEE International Conference on Fuzzy Systems (FUZZ-IEEE) (pp. 1-6). IEEE.

Balci, K., & Salah, A. A. (2015). Automatic analysis and identification of verbal aggression and abusive behaviors for online social games. Computers in Human Behavior, 53, 517-526.

Blackburn, J., & Kwak, H. (2014, April). STFU NOOB! predicting crowdsourced decisions on toxic behavior in online games. In Proceedings of the 23rd international conference on World wide web (pp. 877-888).

Bongaards, T., Adriaanse, M., & Frommel, J. (2024, October). Personalized Matchmaking Restrictions for Reduced Exposure to Toxicity: Preliminary Insights from an Interview Study. In Companion Proceedings of the 2024 Annual Symposium on Computer-Human Interaction in Play (pp. 31-36).

Boustani, K., Tally, A. C., Kim, Y. R., & Nippert-Eng, C. (2020, November). Gaming the name: Player strategies for adapting to name constraints in online videogames. In Proceedings of the Annual Symposium on Computer-Human Interaction in Play (pp. 120-131).

Bovenzi, G. M. (2025). Content moderation in (decentralized) metaverses. Interactive Entertainment Law Review, 1(aop), 1-12.

Brewer, J., Romine, M., & Taylor, T. L. (2020, July). Inclusion at scale: Deploying a community-driven moderation intervention on twitch. In Proceedings of the 2020 ACM designing interactive systems conference (pp. 757-769).

Busch, T., Boudreau, K., & Consalvo, M. (2015). Toxic gamer culture, corporate regulation, and standards of behavior among players of online games. In Video game policy (pp. 176-190). Routledge.

Canossa, A., Salimov, D., Azadvar, A., Harteveld, C., & Yannakakis, G. (2021). For honor, for toxicity: Detecting toxic behavior through gameplay. Proceedings of the ACM on Human-Computer Interaction, 5(CHI PLAY), 1-29.

Castro, D. (2022). Content moderation in multi-user immersive experiences: AR/VR and the future of online speech. Information Technology and Innovation Foundation.

Chawki, M. (2025). AI Moderation and Legal Frameworks in Child-Centric Social Media: A Case Study of Roblox. Laws, 14(3), 29.

Corbett, S. (2019). Computer game licences: The EULA and its discontents. Computer Law & Security Review, 35(4), 453-461.

Cornel, J. A., Pablo, C. C., Marzan, J. A., Mercado, V. J., Fabito, B., Rodriguez, R., ... & De La Cruz, A. (2019, November). Cyberbullying detection for online games chat logs using deep learning. In 2019 IEEE 11th International Conference on Humanoid, Nanotechnology, Information Technology, Communication and Control, Environment, and Management (HNICEM) (pp. 1-5). IEEE.

Cullen, A. L., & Ruberg, B. (2019, August). Necklines and'naughty bits' constructing and regulating bodies in live streaming community guidelines. In Proceedings of the 14th International Conference on the Foundations of Digital Games (pp. 1-8).

de Mesquita Neto, J. A., & Becker, K. (2018). Relating conversational topics and toxic behavior effects in a MOBA game. Entertainment computing, 26, 10-29.

Dolling, J., & Frei, T. (2025, February). AI Voice Chat Moderation Systems in Video Gaming under EU Data Protection Law. In Artificial Intelligence (pp. 71-94). Nomos Verlagsgesellschaft mbH & Co. KG.

Donaldson, S. (2017, January). I predict a riot: Making and breaking rules and norms in league of legends. In Proceedings of DiGRA 2017 Conference.

Fang, A., Yang, W., & Zhu, H. (2023). Shaping Online Dialogue: Examining How Community Rules Affect Discussion Structures on Reddit. arXiv preprint arXiv:2308.01257.

Fiesler, C., Jiang, J., McCann, J., Frye, K., & Brubaker, J. (2018, June). Reddit rules! characterizing an ecosystem of governance. In Proceedings of the International AAAI Conference on Web and Social Media (Vol. 12, No. 1).

Fiesler, C., Lampe, C., & Bruckman, A. S. (2016, February). Reality and perception of copyright terms of service for online content creation. In Proceedings of the 19th ACM conference on computer-supported cooperative work & social computing (pp. 1450-1461).

Figueiredo, C. (2022). Trust and Safety and Fair Play in Video Games: Intentionally Designing Positive Communities. In Game Usability (pp. 215-237). CRC Press.

Fox, J., & Tang, W. Y. (2017). Women’s experiences with general and sexual harassment in online video games: Rumination, organizational responsiveness, withdrawal, and coping strategies. New media & society, 19(8), 1290-1307.

Frommel, J., & Mandryk, R. L. (2023). Individual control over exposure to combat toxicity in games. ACM Games: Research and Practice, 1(4), 1-3.

Frommel, J., & Mandryk, R. L. (2024, May). Toxicity in online games: The prevalence and efficacy of coping strategies. In Proceedings of the 2024 CHI Conference on Human Factors in Computing Systems (pp. 1-12).

Frommel, J., Sagl, V., Depping, A. E., Johanson, C., Miller, M. K., & Mandryk, R. L. (2020, April). Recognizing affiliation: Using behavioural traces to predict the quality of social interactions in online games. In Proceedings of the 2020 CHI conference on human factors in computing systems (pp. 1-16).

Full Citation

Glas, R. (2013). Battlefields of negotiation: Control, agency, and ownership in World of Warcraft (p. 220). Amsterdam University Press.

Grace, T. D., Larson, I., & Salen, K. (2022). Policies of misconduct: A content analysis of codes of conduct for online multiplayer games. Proceedings of the ACM on Human-Computer Interaction, 6(CHI PLAY), 1-23.

Grieman, K. (2019). Lakitu's world: proactive and reactive regulation in video games. Interactive Entertainment Law Review, 2(2), 67-77.

Grimes, S. M. (2007). Terms of service, terms of play in children’s online gaming. The Player’s Realm: Studies on the Culture of Video Games and Gaming, 33-55.

Halbert, D. J. (2009). Public lives and private communities: The terms of service agreement and life in virtual worlds. First Monday.

Epic Games. (2024). Epic Games Transparency Report.  https://safety.epicgames.com/en-US/transparency-reports

Entertainment Arts (2024). EA Transparency Report. https://www.ea.com/news/2024-transparency-report

Ubisoft (2024). Ubisoft Transparency Report. https://www.ubisoft.com/legal/documents/transparencyreport/en-US

XBox (2024). XBox Transparency Report. <https://www.xbox.com/en-US/legal/xbox-transparency-report>

Nintendo (2024). Nintendo Transparency Report.

Niantic (2024). Niantic Transparency Reports.

Take-Two Interactive (2024). Take-Two Transparency Report.

Huth, J., Eichhorn, C., Plecher, D. A., & Pirker, J. (2025, August). Exploring the Potential of an AI-Based Twitch Moderation and Toxicity Detection Bot. In 2025 IEEE Conference on Games (CoG) (pp. 1-4). IEEE.

Jhaver, S., Bruckman, A., & Gilbert, E. (2019). Does transparency in moderation really matter? User behavior after content removal explanations on reddit. *Proceedings of the ACM on Human-Computer Interaction*, *3*(CSCW), 1-27.

Jiang, J., Pan, S., Woods, D. W., & Li, J. (2026). Analyzing Codes of Conduct for Online Safety in Video Games at Scale. *arXiv preprint arXiv:2605.15047*.

Kaiser, E., & Feng, W. C. (2009, November). PlayerRating: a reputation system for multiplayer online games. In 2009 8th Annual Workshop on Network and Systems Support for Games (NetGames) (pp. 1-6). IEEE.

Kocielnik, R., Li, Z., Kann, C., Sambrano, D., Morrier, J., Linegar, M., ... & Alvarez, R. M. (2024). Challenges in moderating disruptive player behavior in online competitive action games. Frontiers in Computer Science, 6, 1283735.

Kou, Y. (2020, November). Toxic behaviors in team-based competitive gaming: The case of league of legends. In Proceedings of the annual symposium on computer-human interaction in play (pp. 81-92).

Kou, Y. (2021). Punishment and its discontents: An analysis of permanent ban in an online game community. Proceedings of the ACM on Human-Computer Interaction, 5(CSCW2), 1-21.

Kou, Y., & Gui, X. (2017). When code governs community.

Kou, Y., & Gui, X. (2021, May). Flag and flaggability in automated moderation: The case of reporting toxic behavior in an online game community. In Proceedings of the 2021 CHI conference on human factors in computing systems (pp. 1-12).

Kou, Y., & Nardi, B. A. (2014). Governance in League of Legends: A hybrid system. FDG, 7(1), 9.

Kou, Y., Ma, R., Zhang, Z., Zhou, Y., & Gui, X. (2024, May). Community Begins Where Moderation Ends: Peer Support and Its Implications for Community-Based Rehabilitation. In Proceedings of the 2024 CHI Conference on Human Factors in Computing Systems (pp. 1-18).

Kou, Y., Zhou, Y., Zhang, Z., & Gui, X. (2024, July). The ecology of harmful design: Risk and safety of game making on a metaverse platform. In Proceedings of the 2024 ACM Designing Interactive Systems Conference (pp. 1842-1856).

Larsson, L., & Johnsson, A. K. (2021). How do the mechanics of honor systems in competitive games facilitate or hinder a toxic game aesthetic?.

Lee, H. S., Lee, H. R., Park, J. U., & Han, Y. S. (2018). An abusive text detection system based on enhanced abusive and non-abusive word lists. Decision Support Systems, 113, 22-31.

Li, Z., Kocielnik, R., Linegar, M., Sambrano, D., Soltani, F., Kim, M., ... & Alvarez, R. M. (2024). Online moderation in competitive action games: How intervention affects player behaviors. arXiv preprint arXiv:2411.01057.

Ma, R., Li, Y., & Kou, Y. (2023, April). Transparency, fairness, and coping: How players experience moderation in multiplayer online games. In Proceedings of the 2023 CHI Conference on Human Factors in Computing Systems (pp. 1-21).

Märtens, M., Shen, S., Iosup, A., & Kuipers, F. (2015, December). Toxicity detection in multiplayer online games. In 2015 International Workshop on Network and Systems Support for Games (NetGames) (pp. 1-6). IEEE.

Modulate AI. (2023). The Impact of AI Voice Moderation on the Call of Duty Player Experience. https://www.modulate.ai/case-studies/modulate-activision-case-study.

Morrier, J., Kocielnik, R., & Alvarez, R. M. (2025). Bandit Algorithms for Efficient Toxicity Detection in Competitive Online Video Games. IEEE Access.

Murnion, S., Buchanan, W. J., Smales, A., & Russell, G. (2018). Machine learning and semantic analysis of in-game chat for cyberbullying. Computers & Security, 76, 197-213.

Neto, J. A., Yokoyama, K. M., & Becker, K. (2017, August). Studying toxic behavior influence and player chat in an online video game. In Proceedings of the international conference on web intelligence (pp. 26-33).

Ng, L. H. X., Lim, A. X. W., & Yoder, M. M. (2025). Modes of toxic behavior and game design considerations in online multiplayer games. First Monday.

Papegnies, E., Labatut, V., Dufour, R., & Linares, G. (2019). Conversational networks for automatic online moderation. IEEE Transactions on Computational Social Systems, 6(1), 38-55.

Patel, J. (2025). Combatting Toxicity: Designing an Intelligent System to Diminish Verbal Harassment in Online Games (Doctoral dissertation, OCAD University).

Poeller, S., & Robinson, R. B. (2024, October). Mute, Block, Punish, Reward? A Call to Shift the Research Focus From Concealing Toxicity in Games to Promoting Genuine Positive Behavior. In Companion Proceedings of the 2024 Annual Symposium on Computer-Human Interaction in Play (pp. 282-284).

Prather, J., Nix, R., & Jessup, R. (2017, June). Trust management for cheating detection in distributed massively multiplayer online games. In 2017 15th Annual Workshop on Network and Systems Support for Games (NetGames) (pp. 1-3). IEEE.

Reid, E., Mandryk, R. L., Beres, N. A., Klarkowski, M., & Frommel, J. (2022). “Bad vibrations”: Sensing toxicity from in-game audio features. IEEE Transactions on Games, 14(4), 558-568.

Reid, E., Mandryk, R. L., Beres, N. A., Klarkowski, M., & Frommel, J. (2022). Feeling good and in control: In-game tools to support targets of toxicity. Proceedings of the ACM on human-computer interaction, 6(CHI PLAY), 1-27.

Sherrick, B., Smith, C., Jia, D. Y. , Kim, J., Woodland, S., &  Fox, J. (2025). How Voice Chat, Cooperativeness, and Competitiveness Impact Prosocial and Antisocial Norms in Multiplayer Online Video Games. *Journal of Broadcasting and Electronic Media*. DOI: 10.1080/08838151.2025.2603246

Saleous, H., & Gergely, M. (2023, November). Uninstall, Noob! Views on rampant toxicity in online gaming. In 2023 IEEE Intl Conf on Dependable, Autonomic and Secure Computing, Intl Conf on

Pervasive Intelligence and Computing, Intl Conf on Cloud and Big Data Computing, Intl Conf on Cyber Science and Technology Congress (DASC/PiCom/CBDCom/CyberSciTech) (pp. 0183-0190). IEEE.

Slovak, P., Salen, K., Ta, S., & Fitzpatrick, G. (2018, April). Mediating conflicts in minecraft: Empowering learning in online multiplayer games. In Proceedings of the 2018 CHI conference on human factors in computing systems (pp. 1-13).

Smith, J., Krasodomski-Jones, A., Olanipekun, M., & Judson, E. (2021). A picture of health: Measuring the comparative health of online spaces. Technical report, Demos.

Sparrow, L. A., Galwey, R., Jovic, D., Hardwick, T., & Butt, M. A. (2024). Towards ethical AI moderation in multiplayer games. Proceedings of the ACM on Human-Computer Interaction, 8(CHI PLAY), 1-30.

Stepanova, N., Muthemba, W., Todrzak, R., Cross, M., Ames, N., & Raiti, J. (2021, June). Natural language processing and sentiment analysis for verbal aggression detection; a solution for cyberbullying during live video gaming. In Proceedings of the 14th PErvasive Technologies Related to Assistive Environments Conference (pp. 117-118).

Steinkuehler, C. (2023). Games as social platforms. *ACM Games Research and Practice*, 1(7), 1-2.

Taveekitworachai, P., Abdullah, F., Gursesli, M. C., Lanata, A., Guazzini, A., & Thawonmas, R. (2024, May). Prompt evolution through examples for large language models–a case study in game comment toxicity classification. In 2024 IEEE International Workshop on Metrology for Industry 4.0 & IoT (MetroInd4. 0 & IoT) (pp. 22-27). IEEE.

Tomkinson, S., & Van Den Ende, B. (2022). ‘thank you for your compliance’: Overwatch as a disciplinary system. Games and culture, 17(2), 198-218.

Toxic Behaviors in Esports Games: Player Perceptions and Coping Strategies

Tseng, Y. S. (2019). The principles of esports engagement: A universal code of conduct?. J. Intell. Prop. l., 27, 209.

Van Hoeyweghen, S. (2024). Speaking of games: AI-based content moderation of real-time voice interactions in video games under the DSA. Interactive Entertainment Law Review, 7(1), 30-46.

Varol, A. C., & Coffee, L. T. (2025, April). Gaming Without Harm: AI-Driven Content Moderation to Improve Safety in Roblox. In 2025 13th International Symposium on Digital Forensics and Security (ISDFS) (pp. 1-4). IEEE.

Vo, H. H. P., Tran, H. T., & Luu, S. T. (2021, August). Automatically detecting cyberbullying comments on online game forums. In 2021 RIVF International Conference on Computing and Communication Technologies (RIVF) (pp. 1-5). IEEE.

Wijkstra, M., Rogers, K., Mandryk, R. L., Veltkamp, R. C., & Frommel, J. (2023, October). Help, my game is toxic! first insights from a systematic literature review on intervention systems for toxic behaviors in online video games. In Companion Proceedings of the Annual Symposium on Computer-Human Interaction in Play (pp. 3-9).

Wijkstra, M., Rogers, K., Mandryk, R. L., Veltkamp, R. C., & Frommel, J. (2024). How to tame a toxic player? A systematic literature review on intervention systems for toxic behaviors in online video games. Proceedings of the ACM on human-computer interaction, 8(CHI PLAY), 1-32.

Woo, J., Park, S. H., & Kim, H. K. (2022). Profane or Not: Improving Korean Profane Detection using Deep Learning. KSII Transactions on Internet & Information Systems, 16(1).

Xiao, S., Jhaver, S., & Salehi, N. (2023). Addressing interpersonal harm in online gaming communities: The opportunities and challenges for a restorative justice approach. ACM Transactions on Computer-Human Interaction, 30(6), 1-36.

Yang, Z., Grenan-Godbout, N., & Rabbany, R. (2023). Towards detecting contextual real-time toxicity for in-game chat. arXiv preprint arXiv:2310.18330.

Yang, Z., Grenon-Godbout, N., & Rabbany, R. (2024). Game on, hate off: A study of toxicity in online multiplayer environments. ACM Games: Research and Practice, 2(2), 1-13.

Yıldırım, N. (2022). The bullying game: sexism based toxic language analysis on online games chat logs by text mining. Journal of International Women's Studies, 24(3), 7.

Yousefi, M., & Emmanouilidou, D. (2021, August). Audio-based toxic language classification using self-attentive convolutional neural network. In 2021 29th European Signal Processing Conference (EUSIPCO) (pp. 11-15). IEEE.

Zhang, Z., Moradzadeh, S., Gui, X., & Kou, Y. (2024). Harmful design in user-generated games and its ethical and governance challenges: An investigation of design co-ideation of game creators on roblox. Proceedings of the ACM on Human-Computer Interaction, 8(CHI PLAY), 1-31.

Zolides, A. (2021). Gender moderation and moderating gender: Sexual content policies in Twitch’s community guidelines. New Media & Society, 23(10), 2999-3015.

Zsila, Á., Orosz, G., & Demetrovics, Z. (2025). Reducing Toxic Behaviors in the Gaming Community: A Social-Psychological Intervention with Lasting Effects. Computers in Human Behavior, 108827.

mova, V., & Bernardino, J. (2013, October). Ethic in massively multiplayer online role-playing games. In 2013 1st International Conference of the Portuguese Society for Engineering Education (CISPEE) (pp. 1-9). IEEE.

Andrigueto, G. R., & Araujo, E. (2020, July). Fuzzy aggressive behavior assessment of toxic players in multiplayer online battle games. In 2020 IEEE International Conference on Fuzzy Systems (FUZZ-IEEE) (pp. 1-6). IEEE.

Balci, K., & Salah, A. A. (2015). Automatic analysis and identification of verbal aggression and abusive behaviors for online social games. Computers in Human Behavior, 53, 517-526.

Blackburn, J., & Kwak, H. (2014, April). STFU NOOB! predicting crowdsourced decisions on toxic behavior in online games. In Proceedings of the 23rd international conference on World wide web (pp. 877-888).

Bongaards, T., Adriaanse, M., & Frommel, J. (2024, October). Personalized Matchmaking Restrictions for Reduced Exposure to Toxicity: Preliminary Insights from an Interview Study. In Companion Proceedings of the 2024 Annual Symposium on Computer-Human Interaction in Play (pp. 31-36).

Boustani, K., Tally, A. C., Kim, Y. R., & Nippert-Eng, C. (2020, November). Gaming the name: Player strategies for adapting to name constraints in online videogames. In Proceedings of the Annual Symposium on Computer-Human Interaction in Play (pp. 120-131).

Bovenzi, G. M. (2025). Content moderation in (decentralized) metaverses. Interactive Entertainment Law Review, 1(aop), 1-12.

Brewer, J., Romine, M., & Taylor, T. L. (2020, July). Inclusion at scale: Deploying a community-driven moderation intervention on twitch. In Proceedings of the 2020 ACM designing interactive systems conference (pp. 757-769).

Busch, T., Boudreau, K., & Consalvo, M. (2015). Toxic gamer culture, corporate regulation, and standards of behavior among players of online games. In Video game policy (pp. 176-190). Routledge.

Canossa, A., Salimov, D., Azadvar, A., Harteveld, C., & Yannakakis, G. (2021). For honor, for toxicity: Detecting toxic behavior through gameplay. Proceedings of the ACM on Human-Computer Interaction, 5(CHI PLAY), 1-29.

Castro, D. (2022). Content moderation in multi-user immersive experiences: AR/VR and the future of online speech. Information Technology and Innovation Foundation.

Chawki, M. (2025). AI Moderation and Legal Frameworks in Child-Centric Social Media: A Case Study of Roblox. Laws, 14(3), 29.

Corbett, S. (2019). Computer game licences: The EULA and its discontents. Computer Law & Security Review, 35(4), 453-461.

Cornel, J. A., Pablo, C. C., Marzan, J. A., Mercado, V. J., Fabito, B., Rodriguez, R., ... & De La Cruz, A. (2019, November). Cyberbullying detection for online games chat logs using deep learning. In 2019 IEEE 11th International Conference on Humanoid, Nanotechnology, Information Technology, Communication and Control, Environment, and Management (HNICEM) (pp. 1-5). IEEE.

Cullen, A. L., & Ruberg, B. (2019, August). Necklines and'naughty bits' constructing and regulating bodies in live streaming community guidelines. In Proceedings of the 14th International Conference on the Foundations of Digital Games (pp. 1-8).

de Mesquita Neto, J. A., & Becker, K. (2018). Relating conversational topics and toxic behavior effects in a MOBA game. Entertainment computing, 26, 10-29.

Dolling, J., & Frei, T. (2025, February). AI Voice Chat Moderation Systems in Video Gaming under EU Data Protection Law. In Artificial Intelligence (pp. 71-94). Nomos Verlagsgesellschaft mbH & Co. KG.

Donaldson, S. (2017, January). I predict a riot: Making and breaking rules and norms in league of legends. In Proceedings of DiGRA 2017 Conference.

Fang, A., Yang, W., & Zhu, H. (2023). Shaping Online Dialogue: Examining How Community Rules Affect Discussion Structures on Reddit. arXiv preprint arXiv:2308.01257.

Fiesler, C., Jiang, J., McCann, J., Frye, K., & Brubaker, J. (2018, June). Reddit rules! characterizing an ecosystem of governance. In Proceedings of the International AAAI Conference on Web and Social Media (Vol. 12, No. 1).

Fiesler, C., Lampe, C., & Bruckman, A. S. (2016, February). Reality and perception of copyright terms of service for online content creation. In Proceedings of the 19th ACM conference on computer-supported cooperative work & social computing (pp. 1450-1461).

Figueiredo, C. (2022). Trust and Safety and Fair Play in Video Games: Intentionally Designing Positive Communities. In Game Usability (pp. 215-237). CRC Press.

Fox, J., & Tang, W. Y. (2017). Women’s experiences with general and sexual harassment in online video games: Rumination, organizational responsiveness, withdrawal, and coping strategies. New media & society, 19(8), 1290-1307.

Frommel, J., & Mandryk, R. L. (2023). Individual control over exposure to combat toxicity in games. ACM Games: Research and Practice, 1(4), 1-3.

Frommel, J., & Mandryk, R. L. (2024, May). Toxicity in online games: The prevalence and efficacy of coping strategies. In Proceedings of the 2024 CHI Conference on Human Factors in Computing Systems (pp. 1-12).

Frommel, J., Sagl, V., Depping, A. E., Johanson, C., Miller, M. K., & Mandryk, R. L. (2020, April). Recognizing affiliation: Using behavioural traces to predict the quality of social interactions in online games. In Proceedings of the 2020 CHI conference on human factors in computing systems (pp. 1-16).

Full Citation

Glas, R. (2013). Battlefields of negotiation: Control, agency, and ownership in World of Warcraft (p. 220). Amsterdam University Press.

Grace, T. D., Larson, I., & Salen, K. (2022). Policies of misconduct: A content analysis of codes of conduct for online multiplayer games. Proceedings of the ACM on Human-Computer Interaction, 6(CHI PLAY), 1-23.

Grieman, K. (2019). Lakitu's world: proactive and reactive regulation in video games. Interactive Entertainment Law Review, 2(2), 67-77.

Grimes, S. M. (2007). Terms of service, terms of play in children’s online gaming. The Player’s Realm: Studies on the Culture of Video Games and Gaming, 33-55.

Halbert, D. J. (2009). Public lives and private communities: The terms of service agreement and life in virtual worlds. First Monday.

Epic Games. (2024). Epic Games Transparency Report.  https://safety.epicgames.com/en-US/transparency-reports

Entertainment Arts (2024). EA Transparency Report. https://www.ea.com/news/2024-transparency-report

Ubisoft (2024). Ubisoft Transparency Report. https://www.ubisoft.com/legal/documents/transparencyreport/en-US

XBox (2024). XBox Transparency Report. <https://www.xbox.com/en-US/legal/xbox-transparency-report>

Nintendo (2024). Nintendo Transparency Report.

Niantic (2024). Niantic Transparency Reports.

Take-Two Interactive (2024). Take-Two Transparency Report.

Huth, J., Eichhorn, C., Plecher, D. A., & Pirker, J. (2025, August). Exploring the Potential of an AI-Based Twitch Moderation and Toxicity Detection Bot. In 2025 IEEE Conference on Games (CoG) (pp. 1-4). IEEE.

Jhaver, S., Bruckman, A., & Gilbert, E. (2019). Does transparency in moderation really matter? User behavior after content removal explanations on reddit. *Proceedings of the ACM on Human-Computer Interaction*, *3*(CSCW), 1-27.

Jiang, J., Pan, S., Woods, D. W., & Li, J. (2026). Analyzing Codes of Conduct for Online Safety in Video Games at Scale. *arXiv preprint arXiv:2605.15047*.

Kaiser, E., & Feng, W. C. (2009, November). PlayerRating: a reputation system for multiplayer online games. In 2009 8th Annual Workshop on Network and Systems Support for Games (NetGames) (pp. 1-6). IEEE.

Kocielnik, R., Li, Z., Kann, C., Sambrano, D., Morrier, J., Linegar, M., ... & Alvarez, R. M. (2024). Challenges in moderating disruptive player behavior in online competitive action games. Frontiers in Computer Science, 6, 1283735.

Kou, Y. (2020, November). Toxic behaviors in team-based competitive gaming: The case of league of legends. In Proceedings of the annual symposium on computer-human interaction in play (pp. 81-92).

Kou, Y. (2021). Punishment and its discontents: An analysis of permanent ban in an online game community. Proceedings of the ACM on Human-Computer Interaction, 5(CSCW2), 1-21.

Kou, Y., & Gui, X. (2017). When code governs community.

Kou, Y., & Gui, X. (2021, May). Flag and flaggability in automated moderation: The case of reporting toxic behavior in an online game community. In Proceedings of the 2021 CHI conference on human factors in computing systems (pp. 1-12).

Kou, Y., & Nardi, B. A. (2014). Governance in League of Legends: A hybrid system. FDG, 7(1), 9.

Kou, Y., Ma, R., Zhang, Z., Zhou, Y., & Gui, X. (2024, May). Community Begins Where Moderation Ends: Peer Support and Its Implications for Community-Based Rehabilitation. In Proceedings of the 2024 CHI Conference on Human Factors in Computing Systems (pp. 1-18).

Kou, Y., Zhou, Y., Zhang, Z., & Gui, X. (2024, July). The ecology of harmful design: Risk and safety of game making on a metaverse platform. In Proceedings of the 2024 ACM Designing Interactive Systems Conference (pp. 1842-1856).

Larsson, L., & Johnsson, A. K. (2021). How do the mechanics of honor systems in competitive games facilitate or hinder a toxic game aesthetic?.

Lee, H. S., Lee, H. R., Park, J. U., & Han, Y. S. (2018). An abusive text detection system based on enhanced abusive and non-abusive word lists. Decision Support Systems, 113, 22-31.

Li, Z., Kocielnik, R., Linegar, M., Sambrano, D., Soltani, F., Kim, M., ... & Alvarez, R. M. (2024). Online moderation in competitive action games: How intervention affects player behaviors. arXiv preprint arXiv:2411.01057.

Ma, R., Li, Y., & Kou, Y. (2023, April). Transparency, fairness, and coping: How players experience moderation in multiplayer online games. In Proceedings of the 2023 CHI Conference on Human Factors in Computing Systems (pp. 1-21).

Märtens, M., Shen, S., Iosup, A., & Kuipers, F. (2015, December). Toxicity detection in multiplayer online games. In 2015 International Workshop on Network and Systems Support for Games (NetGames) (pp. 1-6). IEEE.

Modulate AI. (2023). The Impact of AI Voice Moderation on the Call of Duty Player Experience. https://www.modulate.ai/case-studies/modulate-activision-case-study.

Morrier, J., Kocielnik, R., & Alvarez, R. M. (2025). Bandit Algorithms for Efficient Toxicity Detection in Competitive Online Video Games. IEEE Access.

Murnion, S., Buchanan, W. J., Smales, A., & Russell, G. (2018). Machine learning and semantic analysis of in-game chat for cyberbullying. Computers & Security, 76, 197-213.

Neto, J. A., Yokoyama, K. M., & Becker, K. (2017, August). Studying toxic behavior influence and player chat in an online video game. In Proceedings of the international conference on web intelligence (pp. 26-33).

Ng, L. H. X., Lim, A. X. W., & Yoder, M. M. (2025). Modes of toxic behavior and game design considerations in online multiplayer games. First Monday.

Papegnies, E., Labatut, V., Dufour, R., & Linares, G. (2019). Conversational networks for automatic online moderation. IEEE Transactions on Computational Social Systems, 6(1), 38-55.

Patel, J. (2025). Combatting Toxicity: Designing an Intelligent System to Diminish Verbal Harassment in Online Games (Doctoral dissertation, OCAD University).

Poeller, S., & Robinson, R. B. (2024, October). Mute, Block, Punish, Reward? A Call to Shift the Research Focus From Concealing Toxicity in Games to Promoting Genuine Positive Behavior. In Companion Proceedings of the 2024 Annual Symposium on Computer-Human Interaction in Play (pp. 282-284).

Prather, J., Nix, R., & Jessup, R. (2017, June). Trust management for cheating detection in distributed massively multiplayer online games. In 2017 15th Annual Workshop on Network and Systems Support for Games (NetGames) (pp. 1-3). IEEE.

Reid, E., Mandryk, R. L., Beres, N. A., Klarkowski, M., & Frommel, J. (2022). “Bad vibrations”: Sensing toxicity from in-game audio features. IEEE Transactions on Games, 14(4), 558-568.

Reid, E., Mandryk, R. L., Beres, N. A., Klarkowski, M., & Frommel, J. (2022). Feeling good and in control: In-game tools to support targets of toxicity. Proceedings of the ACM on human-computer interaction, 6(CHI PLAY), 1-27.

Sherrick, B., Smith, C., Jia, D. Y. , Kim, J., Woodland, S., &  Fox, J. (2025). How Voice Chat, Cooperativeness, and Competitiveness Impact Prosocial and Antisocial Norms in Multiplayer Online Video Games. *Journal of Broadcasting and Electronic Media*. DOI: 10.1080/08838151.2025.2603246

Saleous, H., & Gergely, M. (2023, November). Uninstall, Noob! Views on rampant toxicity in online gaming. In 2023 IEEE Intl Conf on Dependable, Autonomic and Secure Computing, Intl Conf on

Pervasive Intelligence and Computing, Intl Conf on Cloud and Big Data Computing, Intl Conf on Cyber Science and Technology Congress (DASC/PiCom/CBDCom/CyberSciTech) (pp. 0183-0190). IEEE.

Slovak, P., Salen, K., Ta, S., & Fitzpatrick, G. (2018, April). Mediating conflicts in minecraft: Empowering learning in online multiplayer games. In Proceedings of the 2018 CHI conference on human factors in computing systems (pp. 1-13).

Smith, J., Krasodomski-Jones, A., Olanipekun, M., & Judson, E. (2021). A picture of health: Measuring the comparative health of online spaces. Technical report, Demos.

Sparrow, L. A., Galwey, R., Jovic, D., Hardwick, T., & Butt, M. A. (2024). Towards ethical AI moderation in multiplayer games. Proceedings of the ACM on Human-Computer Interaction, 8(CHI PLAY), 1-30.

Stepanova, N., Muthemba, W., Todrzak, R., Cross, M., Ames, N., & Raiti, J. (2021, June). Natural language processing and sentiment analysis for verbal aggression detection; a solution for cyberbullying during live video gaming. In Proceedings of the 14th PErvasive Technologies Related to Assistive Environments Conference (pp. 117-118).

Steinkuehler, C. (2023). Games as social platforms. *ACM Games Research and Practice*, 1(7), 1-2.

Taveekitworachai, P., Abdullah, F., Gursesli, M. C., Lanata, A., Guazzini, A., & Thawonmas, R. (2024, May). Prompt evolution through examples for large language models–a case study in game comment toxicity classification. In 2024 IEEE International Workshop on Metrology for Industry 4.0 & IoT (MetroInd4. 0 & IoT) (pp. 22-27). IEEE.

Tomkinson, S., & Van Den Ende, B. (2022). ‘thank you for your compliance’: Overwatch as a disciplinary system. Games and culture, 17(2), 198-218.

Toxic Behaviors in Esports Games: Player Perceptions and Coping Strategies

Tseng, Y. S. (2019). The principles of esports engagement: A universal code of conduct?. J. Intell. Prop. l., 27, 209.

Van Hoeyweghen, S. (2024). Speaking of games: AI-based content moderation of real-time voice interactions in video games under the DSA. Interactive Entertainment Law Review, 7(1), 30-46.

Varol, A. C., & Coffee, L. T. (2025, April). Gaming Without Harm: AI-Driven Content Moderation to Improve Safety in Roblox. In 2025 13th International Symposium on Digital Forensics and Security (ISDFS) (pp. 1-4). IEEE.

Vo, H. H. P., Tran, H. T., & Luu, S. T. (2021, August). Automatically detecting cyberbullying comments on online game forums. In 2021 RIVF International Conference on Computing and Communication Technologies (RIVF) (pp. 1-5). IEEE.

Wijkstra, M., Rogers, K., Mandryk, R. L., Veltkamp, R. C., & Frommel, J. (2023, October). Help, my game is toxic! first insights from a systematic literature review on intervention systems for toxic behaviors in online video games. In Companion Proceedings of the Annual Symposium on Computer-Human Interaction in Play (pp. 3-9).

Wijkstra, M., Rogers, K., Mandryk, R. L., Veltkamp, R. C., & Frommel, J. (2024). How to tame a toxic player? A systematic literature review on intervention systems for toxic behaviors in online video games. Proceedings of the ACM on human-computer interaction, 8(CHI PLAY), 1-32.

Woo, J., Park, S. H., & Kim, H. K. (2022). Profane or Not: Improving Korean Profane Detection using Deep Learning. KSII Transactions on Internet & Information Systems, 16(1).

Xiao, S., Jhaver, S., & Salehi, N. (2023). Addressing interpersonal harm in online gaming communities: The opportunities and challenges for a restorative justice approach. ACM Transactions on Computer-Human Interaction, 30(6), 1-36.

Yang, Z., Grenan-Godbout, N., & Rabbany, R. (2023). Towards detecting contextual real-time toxicity for in-game chat. arXiv preprint arXiv:2310.18330.

Yang, Z., Grenon-Godbout, N., & Rabbany, R. (2024). Game on, hate off: A study of toxicity in online multiplayer environments. ACM Games: Research and Practice, 2(2), 1-13.

Yıldırım, N. (2022). The bullying game: sexism based toxic language analysis on online games chat logs by text mining. Journal of International Women's Studies, 24(3), 7.

Yousefi, M., & Emmanouilidou, D. (2021, August). Audio-based toxic language classification using self-attentive convolutional neural network. In 2021 29th European Signal Processing Conference (EUSIPCO) (pp. 11-15). IEEE.

Zhang, Z., Moradzadeh, S., Gui, X., & Kou, Y. (2024). Harmful design in user-generated games and its ethical and governance challenges: An investigation of design co-ideation of game creators on roblox. Proceedings of the ACM on Human-Computer Interaction, 8(CHI PLAY), 1-31.

Zolides, A. (2021). Gender moderation and moderating gender: Sexual content policies in Twitch’s community guidelines. New Media & Society, 23(10), 2999-3015.

Zsila, Á., Orosz, G., & Demetrovics, Z. (2025). Reducing Toxic Behaviors in the Gaming Community: A Social-Psychological Intervention with Lasting Effects. Computers in Human Behavior, 108827.
